# Economic effects of policy options restricting antimicrobial use for high risk cattle placed in U.S. feedlots

**DOI:** 10.1371/journal.pone.0239135

**Published:** 2020-09-15

**Authors:** Guillaume Lhermie, Pierre Sauvage, Loren William Tauer, Leslie Verteramo Chiu, Karun Kanyiamattam, Ahmed Ferchiou, Didier Raboisson, Harvey Morgan Scott, David R. Smith, Yrjo Tapio Grohn

**Affiliations:** 1 Department of Population Medicine and Diagnostic Sciences, College of Veterinary Medicine, Cornell University, Ithaca, NY, United States of America; 2 IHAP, Université de Toulouse, INRA, ENVT, Toulouse, France; 3 Dyson School of Applied Economics and Management, Cornell SC Johnson College of Business, Cornell University, Ithaca, NY, United States of America; 4 Department of Veterinary Pathobiology, College of Veterinary Medicine and Biomedical Sciences, Texas A&M University, College Station, TX, United States of America; 5 Department of Pathobiology and Population Medicine, College of Veterinary Medicine, Mississippi State University, Starkville, MS, United States of America; University of Illinois, UNITED STATES

## Abstract

The rising public health threat of antimicrobial resistance, the influence of food service companies, as well as the overall lack of positive image of using medical products in intensive farming are major drivers curbing antimicrobial use. In the future, government policies may affect practices of antimicrobial use in beef production in feedlots, a prominent current user of antimicrobials in animal agriculture, but also the agricultural industry generating the highest cash receipt in the U.S. Our objective was to estimate the cost effect from the following policies in feedlots: 1) using antimicrobials for disease prevention, control, and treatment; 2) using antimicrobials only for treatment of disease; and 3) not using antimicrobials for any reason. We modelled a typical U.S. feedlot, where high risk cattle may be afflicted by diseases requiring antimicrobial therapy, namely respiratory diseases, liver abscesses and lameness. We calculated the net revenue loss under each policy of antimicrobial use restriction. With moderate disease incidence, the median net revenue loss was $66 and $96 per animal entering the feedlot, for not using antimicrobials for disease prevention and control, or not using any antimicrobials, respectively, compared to using antimicrobials for disease prevention, control, and treatment. Losses arose mainly from an increase of fatality and morbidity rates, almost doubling for respiratory diseases in the case of antimicrobial use restrictions. In the case of antimicrobial use prohibition, decreasing the feeder cattle price by 9%, or alternatively, increasing the slaughter cattle price by 6.3%, would offset the net revenue losses for the feedlot operator. If no alternatives to antimicrobial therapy for prevention, control and treatment of current infectious diseases are implemented, policies that economically incentivize adoption of non-antimicrobial prevention and control strategies for infectious diseases would be necessary to maintain animal welfare and the profitability of beef production while simultaneously curbing antimicrobial use.

## Introduction

Cattle production is the most important agricultural industry in the U.S., accounting in 2019 for $68 billion of the $374 billion of U.S. agricultural commodity cash receipts [1]. Approximately 11 million tonnes of beef are harvested every year, comprising culled dairy and beef cows and bulls, and feeder calves either finished on grass or in feedlots [2]. Feedlots are concentrated feeding operations, where weaned steers and heifers are grouped and finished with energy-dense rations, for periods of 100 to 300 days, depending on placement weight, and feeding conditions. In the context of a growing global demand for meat, feedlot operators must maintain or even improve their productivity. Stress generated by weaning, transport from cow-calf or backgrounding operations to feedlots, as well as commingling, constitute risk factors for infectious respiratory diseases, occurring mainly in the first weeks after arrival to the feedlot. Diseases afflicting cattle diminish the efficiency of the production process [3]. First, diseases may decrease the output sold, by increasing animal losses due to involuntary culling or mortality. Second, diseases may decrease the efficiency of production factors, leading for example to an increase in the feeding period, or a decrease in feed conversion. Third, disease generates additional expenditures for treatments.

Beef feedlot operators commonly use antimicrobials (AM) to limit the impacts of infectious diseases. In the remainder of the manuscript, we use the term antimicrobials interchangeably with antibiotics i.e., compounds having an antibacterial activity. Bovine Respiratory Disease (BRD) is by far the most frequently occurring clinical disease, affecting up to 36% of cattle placed on feed [4]. Antimicrobials are used routinely for BRD prevention and control [5, 6]. One of the current AMU practices applied to pens of cattle considered at high risk for BRD consists of treating the entire cohort of animals on arrival with a long-acting AM, to reduce the incidence or prevent the appearance of clinical signs. Current treatments include macrolides, phenicols, tetracyclines, sulfonamides and cephalosporins [7, 8].

Liver abscesses (LA) are localized infections caused by anaerobic bacteria. It is generally recognized that these infections are consequences of rumenitis and ruminal acidosis, generated by ramping up of high-grain feeding [9]. The prevalence of LA at slaughter averages from 10 to 20% in most feedlots [10]. The control of LA is based on in-feed use of AM that have been approved for LA prevention. Currently in the U.S., 6 AM are indicated for the prevention of LA; namely, bacitracin, chlortetracycline, oxytetracycline, neomycin, tylosin and virginiamycin. Tylosin is by far the most commonly used [11].

Infectious causes of lameness in feedlot cattle include foot rot and arthritis, due to several bacteria species. Lameness has a negative impact on animal welfare, as it causes pain and reduces social interactions, and may consequently decrease feed consumption [12]. Data from western U.S. feedlots showed that lameness accounted for 16% of health problems and 5% of fatalities [13]. Prevention of lameness is essentially based on good hygiene, feeding and housing, and includes nutritional supplements such as zinc. Curative parenteral treatments include AMU: e.g., ceftiofur, oxytetracycline, or florfenicol [14].

The most up to date data on antimicrobial use for treatment, control, and prevention are limited to irregular NAHMS reporting (last report dated 2011) which tends to ask all-or-none questions regarding use. Complementary to these, updated FDA reports on annual sales of antibiotic classes requests sponsors to estimate which livestock species their products are destined for. Ceftiofur and enrofloxacin each were introduced in the late 1980s. Subsequent labels for the ceftiofur molecule into longer duration formulations were undertaken to extend product self-life and to expand labels to include control (metaphylaxis) indications where a single dose was necessitated for treating large numbers of animals. Tilmicosin and oxytetracycline (also from pre-1990s) were earlier such formulations. Lately, the longer duration macrolide products such as tulathromycin and gamithromycin have (anecdotally) supplanted Ceftiofur Crystalline-Free Acid (CCFA–long duration ceftiofur) for BRD control. Ceftiofur Crystalline-Free Acid has a 13-day slaughter withholding period. On the other hand, tulathromycin has a much longer withholding period (and cannot be used in adult dairy cattle). While ceftiofur remains widely used in dairy production (owing to its zero-day milk withhold) the macrolides (including tilmicosin) are the majority of BRD control metaphylaxis products used at present in beef [15]

Quantitative data on AMU in feedlots are sparse; however, aggregate data from the U.S. Food and Drug Administration (FDA) have shown that dairy and beef cattle accounted for approximately 50% of non-medically and medically important AM (in tons of active ingredients) in food animal production [16]. It is noteworthy that macrolides and 3^rd^ generation cephalosporins, which are used in feedlots, are classified as highest-priority critically important AM for human medicine by the World Health Organization, being both (i) used to treat infections caused by bacteria possibly transmitted from non-human sources, or with resistance genes from non-human source, as well as (ii) sole, or one of limited available therapies, to treat serious bacterial infections in people [17]. As the efficacy of AM at controlling bacterial diseases is high, their use clearly enhances overall animal productivity; as such, they remain a widely used tool [18]. However, the increasing evidence of the contribution of AMU in animal agriculture to the public health threat of antimicrobial resistance (AMR) has emphasized the paramount importance of prudent AMU in food animal production. Indeed, AMU in cattle production leads to selection of resistant bacteria from the commensal and pathogenic enteric microflora of animals, potentially transmitted to humans [19]. To our knowledge, the quantitative impact of AMU in cattle production on AMR in humans has not been measured. Yet consumers, businesses and public advocacy organizations have demanded a reduction of AMU in animal agriculture. In an international context where initiatives aiming to curb AMR are flourishing, it is possible that global policies on AMU will affect U.S. beef production through trade restrictions. Our objective is therefore to evaluate the economic impact of different policies for AMU in feedlots in the U.S., using a partial budgeting method. We compared three alternative scenarios 1) using AM for disease prevention, control, and treatment (PCT); 2) not using AM for prevention or control (No-PC); and 3) not using AM for any reason (No-PCT).

## Material and methods

We used a simulation model built in Microsoft Excel (Microsoft, Redmond, WA, USA), representative of a 100 head pen of a large U.S. feedlot feeding high risk cattle to estimate the average net costs and benefits of AMU restrictions in beef production using a partial-budgeting approach. Sensitivity analysis was then performed to determine the impact of various biologic and economic parameters of the model.

### Scenario definitions

#### Prevention, Control, and Treatment uses of AM scenario (PCT)

In the PCT scenario, AM are administered to all cattle in the pen at or shortly after arrival, primarily as a strategy for preventing or controlling bacterial pneumonia as well as liver abscesses. In prevention, AM are used before the onset of disease in the population, but given its likelihood of apparition, and under veterinary prescription. In control, AM are given to a group of animals to mitigate the impact of disease already observed in some animals of the group. In treatment, AM are used only to treat individuals with clinical signs of diseases. Subsequently, observed clinical cases of illness are treated individually. This scenario typically occurs under the oversight and direction of veterinarians because the AM most commonly used are either prescription medications or require an order from a veterinarian (Veterinary Feed Directive) in the case of AM delivered in feed. We assumed that antimicrobial treatment was implemented for cases of observed disease. Using an ionophores medication was still possible in this scenario.

#### Producing without antimicrobials: No-PCT scenario

Under the No-PCT scenario, recourse to therapeutic AMU was prohibited for prevention, control or treatment of disease. Using ionophores was still possible.

#### Limiting the use of antimicrobials to curative treatments: No-PC scenario

Under the No-PC scenario, AM are only used to treat animals displaying clinical signs of diseases.

### Feedlot model parameters

#### Production process characteristics of healthy animals

In each scenario, we modeled a generic pen of 100 steers at high risk to contract infectious diseases, averaging 295 kg at arrival and slaughtered at 590 kg (Table 1). The pen was considered as a closed system, with animals not displaying clinical signs at the beginning of the feeding period. Apart from AMU, other production methods were kept similar between the different scenarios. In the PCT scenario, the feeding period was assumed to be 197 days, divided into two successive first (P1) and second (P2) periods: a P1 of 37 days and a P2 of 160 days. The average daily gain (ADG) was 0.97 kg/d and 1.63 kg/d in P1 and P2, with an ADG of 1.50 kg/d over the feeding period. Because the steers did not receive prophylactic AM in the No-PC and No-PCT scenarios, we assumed, based on estimates found in the literature, that the ADG was lower in those scenarios (P1: 0.90 kg/d; P2: 1.52 kg/d; whole feeding period: 1.40 kg/d) and that the feeding period was longer (P1: 40 d; P2: 171 d; whole feeding period: 211 d) [20–22].

**Table 1:** Production characteristics for a healthy steer used the model parametrization.

Production characteristics	Scenario			References
	PCT	No-PC	No-PCT	
**Entering weight**	295 kg	295 kg	295 kg	20, 21, 22
**Slaughter weight**	590 kg	590 kg	590 kg	
**Average ADG, PCT**	1.5 kg/d	1.4 kg/d	1.4 kg/d	
**ADG, P1**	0.97 kg/d	0.9 kg/d	0.9 kg/d	
**ADG, P2**	1.63 kg/d	1.52 kg/d	1.52 kg/d	
**P1 duration**	37 d	40 d	40 d	
**P2 duration**	159 d	171 d	171 d	
**Feeding period duration**	197 d	211 d	211 d	

ADG: Average Daily Gain; PCT: Prevention, Control, and Treatment; No-PCT: no Prevention, no Control, and no Treatments; No-PC: no Prevention, no Control; P1: Period 1; P2: Period 2.

Consistent with Wileman et al. [23], the ADG of healthy animals that received AM as prophylaxis for BRD was 7.3% greater than the ADG of animals that did not receive prophylaxis.

Daily production costs were calculated according to Lawrence and Ellis [24]. They reported feeding and operating costs (labor, manure handling, equipment, and interest). We calculated the feeding costs according to the feeding ration described by Lawrence and Ellis [24] and the 3 year (2016–2018) average prices of corn, alfalfa hay and 50% dry-matter distillers’ grains [25]. We assumed veterinary costs (for implants, pest control and vaccination) of $19.44 per animal for the entire feeding period [24, 26, 27]. Transportation costs were also included. Production costs are reported in Table 2.

**Table 2:** Production costs for model parametrization. Antimicrobial costs are applicable only in prevention and control.

Parameter name	Mean	SD	Min	Max	References
**Feeder cattle price ($/kg)**	3.44	0.3	2.69	4.04	30
**Daily Feed cost per head ($)**	1.63	-	1.17	2.73	24, 25
**Daily operating costs ($)**	0.38	-	-	-	24, 26, 27
**Transportation cost per head($)**	13.99	-	-	-	
**Implant per head ($)**	12	-	-	-	
**Vaccination per head ($)**	7.44	-	-	-	
**Antimicrobials per head ($)**	20	-	-	-	28, 29
**Slaughter cattle price ($/kg)**	2.62	0.20	2.23	3.00	20, 30

In the PCT scenario, AM were used in feed or parenterally for prevention and control of bovine respiratory diseases (BRD) and liver abscesses (LA). Costs of antimicrobial prevention and control were set at $20 per head [28, 29].

The average feeder cattle price was set at $3.44 per kg. (i.e., as average of the 295 kg-non-fed steer price from January 2016 to December 2018) [30].

#### Diseases

Three main categories of diseases were included in our model, each of them divided into subcategories.

The first category consisted of bovine respiratory disease. We assumed that BRD could be observed (i.e., steers present clinical signs of disease) during P1 and P2. In addition, BRD affected some steers subclinically. Liver abscesses comprised the second category. Consistent with the literature, we assumed that three grades of liver abscesses could be observed at slaughter: LA- (one or two small abscesses or presence of abscess scars; LA (two to four well-organized abscesses); and LA+ (one or more large abscesses or multiple small active abscesses) [10, 31]. The third category, lameness, was divided into two subcategories: foot rot (Lame-FR) and infectious arthritis (Lame-IA).

We assumed an average incidence for each disease and each scenario.

In the PCT scenario, the incidence of disease in the pen was broken into low, moderate, and high, with an average incidence risk estimated in each category of disease (See Table 3) in accordance with the mean incidence rates reported in several studies [10, 12, 22, 31–37, 38].

**Table 3:** Cumulative incidence estimates of diseases for model parametrization.

Disease	Low incidence (%)		Moderate incidence (%)		High incidence (%)		References
	PCT	Others	PCT	Others	PCT	Others	
**BRD-Cl1**	6.4	12.3	12.0	23.1	18.4	35.4	22, 28, 33, 34, 35, 36, 37
**BRD-Cl2**	1.6	3.1	3.0	5.8	4.6	8.8	
**BRD-SubCl**	10.0	19.2	20.0	38.5	30.0	57.7	
**LA-**	4.5	16.7	8.5	31.5	12.0	44.4	10, 31, 32
**LA**	2.3	8.5	6.0	22.2	9.0	33.3	
**LA+**	4.5	16.7	10.0	37.0	22.0	81.5	
**Lame-FR**	0.1	0.1	1.5	1.5	13.0	13.0	12, 13, 38
**Lame-IA**	0.1	0.1	0.5	0.5	7.0	7.0	

PCT: Prevention, Control and Treatment scenario; BRD-Cl1: Bovine Respiratory Disease with clinical signs occurring in the period 1; BRD-Cl2: Bovine Respiratory Disease with clinical signs occurring in the period 2; BRD-SubCl: Bovine Respiratory Disease with subclinical signs; LA-: Liver Abscess, mild intensity, LA: Liver Abscess, moderate intensity; LA+: Liver Abscess, severe intensity; Lame-FR: Footrot; Lame-IA: Infectious Arthritis.

Prophylactic AMU decreased the incidence of diseases against which an AM is administered, if pathogens were susceptible to the administered AM. The disease incidence rates in No-PC and No-PCT were similar, and were calculated with the following formula, where RR_MORB_ was the relative risk of morbidity when AM are used in prophylaxis:

$$INCIDENCE_{No‐PC}=INCIDENCE_{PCT}/RR_{MORB}.$$

The relative risks of morbidity were equal to 0.52 [39] for respiratory diseases and 0.27 [23] for LA. We did not find published literature concerning the efficacy of prophylactic therapy against foot rot and infectious arthritis. Thus, we set these relative risks to 1.

### Evaluation of the impacts of infectious diseases

The cumulated impact of each disease was estimated per sick animal, according to the following components: realizer rate, fatality rate, number of days on feed until slaughter (DOF), quality grade (QG) (% of sick animals having a grade loss), treatment costs ($), and labor costs ($). Realizers were cattle that failed to respond to treatment. The realizer rate is the proportion of cattle marketed early because of morbid condition. Case fatality rate consists of the proportion of sick animals that died from the disease. We assumed that diseases were independent from each other, and that they could occur only once.

For the PCT scenario, estimates of impacts provided by previous research were generally extracted from studies in which AM were used to manage infectious diseases [22, 23, 31, 32, 34–36, 39–43]. Therefore, we used an average value from the literature for each cost impact component, assuming that AM were used to achieve these values.

For the No-PC scenario, we calculated the realizer rate and the case fatality rate using the relative risks (RR) or odds ratios found in the literature [39] according to the following formulas:

$$Realizer\;rate_{No‐PC}=RR_{realizer\;rate}\;*\;realizer\;rate_{PCT}$$

$$Case\;fatality\;rate_{No‐PC}=RR_{fatality\;rate}\;*\;case\;fatality\;rate_{PCT}$$

These relative risks were set to one for subclinical diseases and when no data were found in the literature.

In the No-PCT scenario, for each disease, we set intermediate values for case fatality rate and realizer rate, between those used in the No-PC scenario and the highest estimates found in the literature.

We set an average value of ADG in each scenario, and allowed ADG to vary in a range of ± 15% of the average in the model. We assumed that the impacts of each disease on ADG were consistent between the PCT and No-PC scenarios. In the No-PCT scenario, for each disease, the disease impact on ADG was intermediate between the value used in the No-PC scenario and the highest value found in the literature. Consistent with the literature, the loss of ADG in the case of BRD was applied to the entire duration of the feeding phase concerned [32, 34, 35]. In the case of liver abscesses, the loss of ADG was applied from day 0. In the case of lameness, the loss of ADG (i.e., foot rot and infectious arthritis) was applied from the onset of the disease [37]. The average day of onset of disease, day of death and day of realizer slaughter are reported in Table 4.

**Table 4:** Average day of onset, day of death and day of anticipated slaughter, for each infectious disease.

Disease	Onset of disease (days)	Average day of death after entering feedlot	Average day of realizer slaughter after entering feedlot
**BRD-Cl1**	20	50	50
**BRD-Cl2**	83	113	113
**Lame-FR**	85	101	118
**Lame-IA**	32	48	65

BRD-Cl1: Bovine Respiratory Disease with clinical signs occurring in Period 1; BRD-Cl2: Bovine Respiratory Disease with clinical signs occurring in Period 2; Lame-FR: Foot rot; Lame-IA: Infectious Arthritis.

For modeling purposes, we held constant for healthy cattle the slaughter weight of 590 kg, and therefore assumed that diseases would decrease the ADG, and consequently increased the number of days on feed. Thus, the impact of diseases on ADG was converted and expressed as additional days on feed (Extra-DOF).

The slaughter cattle selling price depends on Yield and Quality Grades of the carcass. The base price is generally defined for the Quality Grade “Choice” and the Yield Grade “3”. In our model, we assumed that healthy animals reached this level. Diseases can affect the grades of a certain percentage of animals that are slaughtered at a regular slaughter weight. We assumed that 10% of steers presenting clinical diseases and 2% of steers affected by subclinical diseases had a loss of quality grade [33, 44]. As several studies have reported that diseases did not impact the Yield Grade enough to induce a penalty, we did not include the Yield Grade in the model [31, 33, 44, 45].

Additional treatment costs and additional labor costs per head were set at constant values for each scenario, and their total costs varied only according to the incidence of disease. We assumed that all sick animals were treated in the No-PC and PCT scenarios. Only treated animals required additional labor.

For simplification purposes, each disease was modeled at the individual level as only occurring once and each was considered independent from the other diseases. All parameter estimates extracted from available literature are shown in Table 5.

**Table 5:** Default disease impacts for model parametrization.

Impact of disease																		
Disease	Extra-DOF (d)			Loss-QG (% of animals)			Fatality rate (%)			Realizer rate (%)			Cost of treatment / sick animal ($)			Labor cost / sick animal ($)		
	PCT	No-PC	No-PCT	PCT	No-PC	No-PCT	PCT	No-PC	No-PCT	PCT	No-PC	No-PCT	PCT	No-PC	No-PCT	PCT	No-PC	No-PCT
**BRD-Cl1**	3	3	6	10	10	10	4.7	7.6	14.7	5	5	5	20	20	0	1.18	1.18	0
**BRD-Cl2**	0	0	0	10	10	10	4.7	7.6	14.7	5	5	5	20	20	0	1.18	1.18	0
**BRD-SubCl**	10	11	14	2	2	2	0	0	0	0	0	0	0	0	0	0	0	0
**LA-**	4	4	7	2	2	2	0	0	0	0	0	0	0	0	0	0	0	0
**LA**	11	12	16	2	2	2	0	0	0	0	0	0	0	0	0	0	0	0
**LA+**	19	21	23	2	2	2	0	0	0	0	0	0	0	0	0	0	0	0
**Lame-FR**	2	3	13	10	10	10	7.2	7.2	12.1	3.1	3.1	4.2	12	12	0	2	2	0
**Lame-IA**	165	179	179	10	10	10	22	22	30.8	4.5	4.5	5.8	17	17	0	2	2	0

Extra-DOF: Additional Days On Feed; Loss-QG: Loss of Quality Grade; BRD-Cl1: Bovine Respiratory Disease with clinical signs occurring in Period 1; BRD-Cl2: Bovine Respiratory Disease with clinical signs occurring in Period 2; BRD-SubCl: Bovine Respiratory Disease with subclinical signs; LA-: Liver Abscess, mild intensity, LA: Liver Abscess, moderate intensity; LA+: Liver Abscess, severe intensity; Lame-FR: Foot rot; Lame-IA: Infectious Arthritis. PCT: Prevention Control and Treatment; No-PC: no Prevention, no Control; No-PCT: no Prevention, no Control and no Treatment.

### Costs and revenues calculation

#### Cost and revenue estimates

The impacts were estimated for a pen of 100 steers with average incidence rates of disease.

First, an average cost was estimated for each cost component. The calculation method of each cost component was similar for each disease.

*Lost revenues*. The lost revenue of a dead animal was set as the gross revenue of a healthy animal at slaughter. The lost revenue of a realizer was calculated by subtracting the gross revenue of a realizer from the gross revenue of a healthy steer at slaughter.

The gross revenue was calculated as the product of weight (kg) at slaughter times the selling price/kg. To calculate the gross revenue, we used an average slaughter price of $2.62 /kg, corresponding to a 3-year average of the selling price of live-fed animals [20, 30].The selling price of live realizer was equal to 53% of the price of animals that reach the optimal slaughter weight (590 kg) [13, 37].

The loss of Quality Grade, from Choice to Select or Standard for example, induces about a 10% price penalty [46]. We used this value to calculate the impact of loss of Quality Grade due to disease incidence.

*Additional costs and cost adjustments*. We calculated the daily production costs (sum of feed and daily operating costs) for a healthy animal. We calculated the costs of treatment and labor costs for a sick animal, and multiplied the values by the number of sick animals under each scenario and incidence (low, moderate, high) rate. For each clinical disease, we set an average day of death or realizer slaughter according to published data [12, 36], and calculated the number of days for which a steer should have been fed until slaughter (Table 3). The weight of realizer and the number of days on feed were calculated. Additional days on feed only affected sick animals that recovered and were slaughtered at optimal weight (590 kg). We then multiplied this number by the daily production costs, to obtain the additional (or lower) costs of finishing a previously diseased animal, assuming the animal recovered sufficiently from the disease event in order to gain optimal weight.

For each scenario for the three incidence rates, the net revenue was calculated at the pen level by subtracting the costs and the lost revenue from the gross revenue.

#### Scenarios analysis

We calculated the net impact by subtracting the net revenues of the No-PC and No-PCT scenarios from the net revenue of the PCT scenario.

For the prices of purchase and sale of steers, we calculated the variations in feeder cattle prices and slaughter prices, all other things being equal, required for the feedlot to be indifferent between the PCT scenario and the alternative scenarios. To do so, we modelled the net revenue as a function of the feeder price or the slaughter cattle price, for each scenario and each level of disease incidence. Then, we calculated required feeder and slaughter cattle prices to equalize the net revenue in each scenario.

#### Sensitivity analysis

Sensitivity analyses were performed using the @Risk (Palisade Corporation, Ithaca, NY, USA) Excel add-in, to determine the influence of stochastic and deterministic input parameters on outcome values. Input components (feed costs, relative risks, AM effects and costs, and feeder cattle prices) and output components (slaughter price) were modeled stochastically. The results are presented in a regression tornado diagram, which depicts the change in the dependent variable when the independent variable increases by one SD with all other variables held constant. The @Risk Excel add-in runs a multiple regression analysis for each iteration with the dependent variable of interest (the net impact) and the simulated values of each stochastic independent variables [47].

## Results

### Net impact under No-PCT and No-PC scenarios over PCT scenario

**Fig 1:**
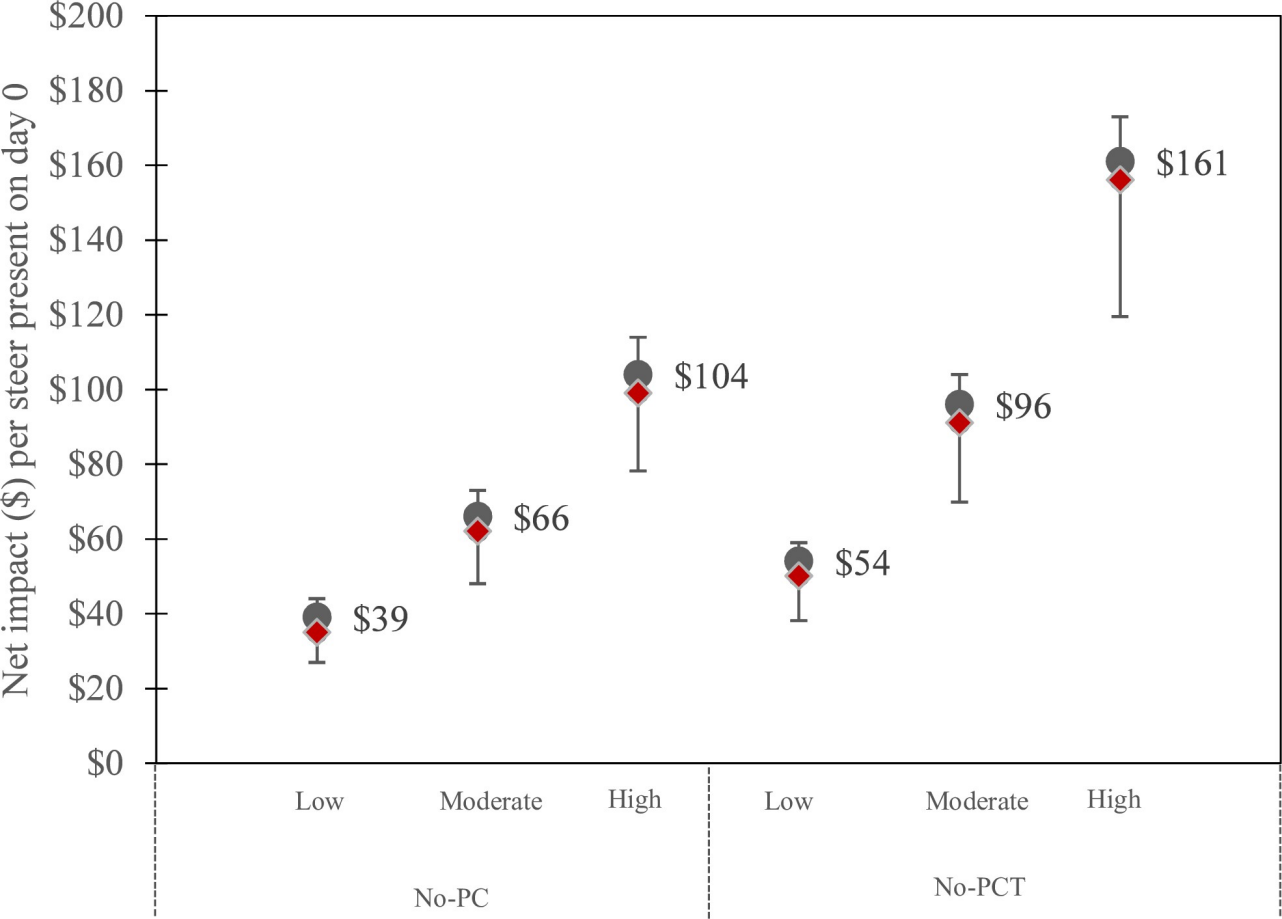
Net impact, per steer present in the feedlot at day 0, for the No-PC and No-PCT scenarios, compared with the PCT scenario, as a function of the level of disease incidence. The grey dots represent the median value, written on the right side, the red diamonds represent the mean, and the bars represent the 25^th^ and 75^th^ percentiles values. PCT: Prevention Control and Treatment; No-PC: no Prevention, no Control; No-PCT: no Prevention, no Control and no Treatment.

Estimates of net impact under the No-PCT scenario and under the No-PC scenario are presented in Fig 1. In a situation of moderate incidence of diseases, the median net impact per steer entering the feedlot over the PCT scenario was $66 (25^th^ percentile: 61, 75^th^ percentile: 73) and $96 (25^th^ percentile: 89, 75^th^ percentile: 104), in the No-PC and No-PCT scenarios, respectively. The net impact increased with the incidence of diseases.

**Fig 2:**
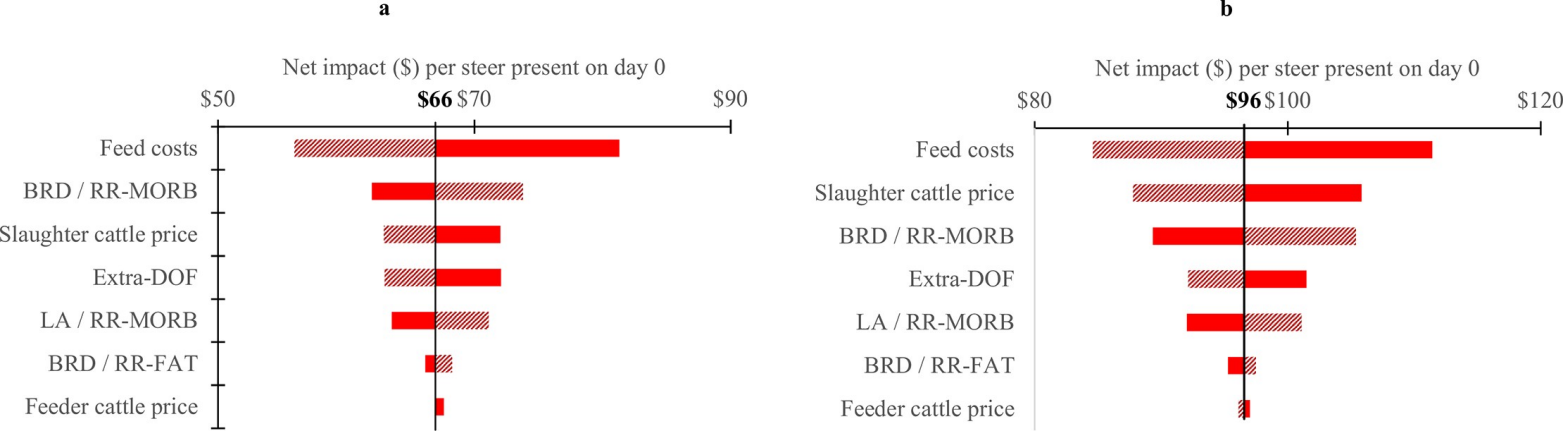
Tornado diagram for factors influencing the net impact (median value) in moderate incidence. Fig 2A: No-PC scenario compared to PCT scenario; Fig 2B: No-PCT scenario compared to PCT scenario. Solid (positive) or dashed (negative) bars indicates the sign of coefficient For example, a positive coefficient e.g., Feed costs with solid bar extending to the right, indicates that this input has a positive impact: increasing this input will increase the net impact. The values (in bold) are median values from Fig 1. PCT: Prevention Control and Treatment; No-PC: no Prevention, no Control; No-PCT: no Prevention, no Control and no Treatment; BRD / RR MORB: relative risk of Bovine Respiratory Disease morbidity when antimicrobials are not used for prevention. LA / RR MORB: relative risk of liver abscesses morbidity when antimicrobials are not used for prevention; BRD / RR FAT: relative risk of Bovine Respiratory Disease fatality when antimicrobials are not used for prevention; Extra-DOF: Additional Days On Feed.

Sensitivity analysis showed that the net impact was most influenced by feed costs, followed by the relative risk of morbidity when AM are used in prevention and control (RR_MORB_), slaughter cattle price and extra days on feed, when comparing the No-PC over the PCT scenario (Fig 2A). The net impact was most influenced by feed costs, followed by slaughter cattle price, the relative risk of morbidity when AM are used in prevention and control (RR_MORB_), and extra days on feed, when comparing the No-PCT over the PCT scenario (Fig 2B). Regardless of the scenario, cattle prices and feed costs were the two most important factors influencing the net impact that were not under the direct control of feedlot operators.

### Additional costs and lost revenue imputable to diseases under each scenario

**Fig 3:**
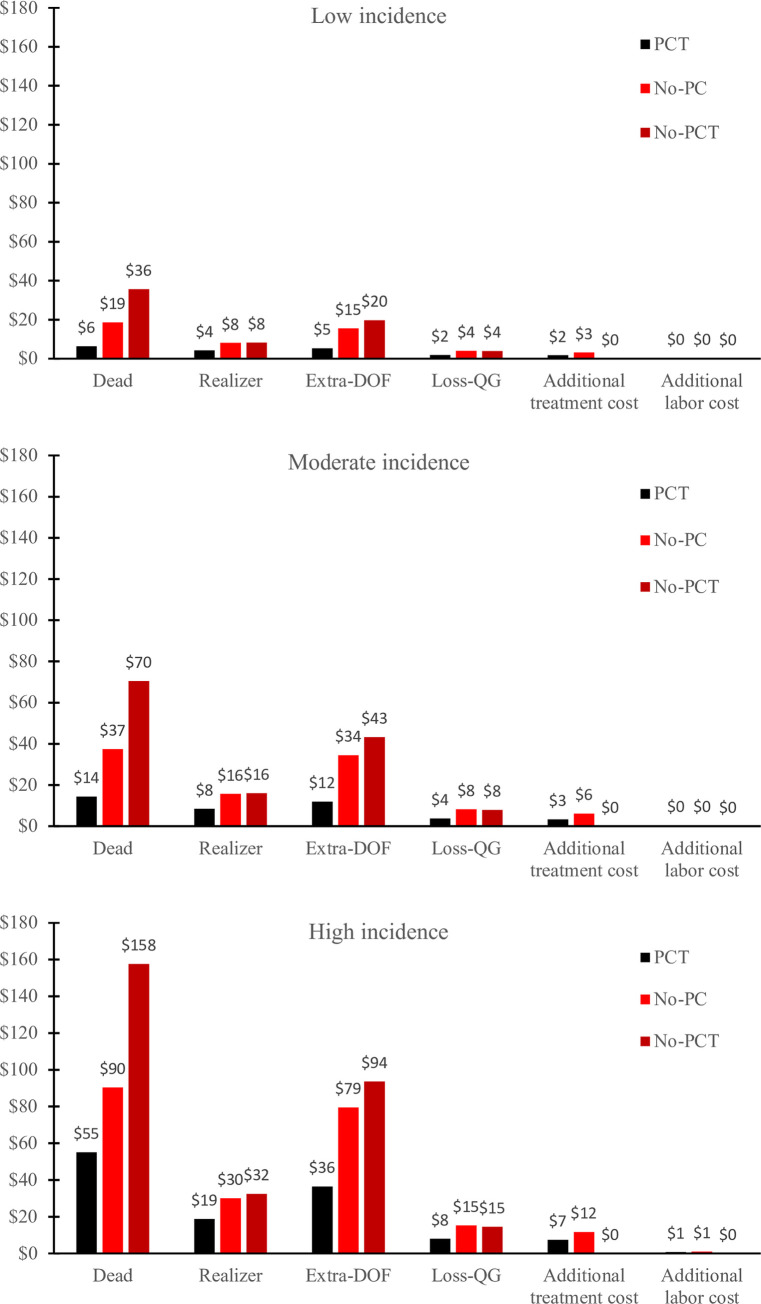
Additional mean costs and lost revenues per steer present in the feedlot at day 0, for the PCT, No-PC and No-PCT scenarios. Mean estimates were used to parameterize the model for each level of incidence. PCT: Prevention Control and Treatment; No-PC: no Prevention, no Control; No-PCT: no Prevention, no Control and no Treatment; Extra-DOF: Additional Days On Feed; Loss-QG: Loss of Quality Grade.

Costs and lost revenue under the PCT scenario, the No-PCT scenario and under the No-PC scenario are presented in Fig 3. In a situation of moderate incidence of diseases, the sum of additional costs and lost revenue per steer were $42, $102, $137 in the PCT, No-PCT and No-PC scenarios, respectively. Breaking down additional costs and lost revenues per parameter shows that mortality and additional days on feed were the largest cost components (Fig 3).

### Indifference between scenarios

To produce equal net revenue under the No-PC scenario or No-PCT scenarios compared to the PCT scenario, feedlot operators would have to purchase steers between 3.5% to 9.7%, and 4.9% to 15.4% respectively, lower than the average purchase price, depending on the incidence of diseases. Importantly, these lower prices directly affect primary cow-calf producers upstream in the production system. Alternatively, if feedlot steers could sell between 2.3% to 7%, and 3.3% to 11.6% higher than the average slaughter price, respectively, they would be indifferent to a change of scenario. The results are depicted in Table 6.

**Table 6:** Variations in feeder cattle prices and slaughter prices required for indifference between PCT, No-PCT and No-PC scenarios.

	No-PC scenario			No-PCT scenario		
Incidence level	Low	Moderate	High	Low	Moderate	High
	Mean	Mean	Mean	Mean	Mean	Mean
	[min-max]	[min-max]	[min-max]	[min-max]	[min-max]	[min-max]
Feeder price (Δ% from PCT)	-3.5	-6.1	-9.7	-4.9	-9.0	-15.4
	[-17.8 –+23.5]	[-20.0 - +20.1]	[-23.0 - +15.5]	[-18.9 - +21.7]	[-22.4 - +16.5]	[-27.9 - +8.3]
Slaughter cattle price (Δ% from PCT)	2.3	4.2	7.0	3.3	6.3	11.6
	[-10.8 - +20.0]	[-9.1 - +22.2]	[-6.7 - +25.5]	[-9.9 - +21.2]	[-7.3 - +24.7]	[-26.4 - +31.0]

PCT: Prevention Control and Treatment; No-PC: no Prevention, no Control; No-PCT: no Prevention, no Control and no Treatment.

## Discussion

The objective of our study was to evaluate the economic impact of different policies for AMU in feedlots in the U.S., using a partial budgeting method calibrated with data from U.S. and Canadian feedlots. We evaluated the impact of restrictions regarding therapeutic uses of antimicrobials only. We compared each of our alternative scenarios with a baseline scenario (PCT), mimicking the current practices of antimicrobial therapy. In addition, we incorporated in our model the major diseases reported in feedlot operations. We evaluated such impact for cattle placed in large feedlots (>1,000 head capacity) in pens of 100 heads, as they represented 81% of the cattle placed in the U.S. in 2019 [48]. We focused on high risk cattle, as the adoption of programs aiming to lower diseases’ risk, such as preconditioning programs, is still low in the U.S. [49, 50].

In the PCT scenario and with moderate disease incidence, the average cost of a clinical case of BRD was $138 and the net revenue obtained from a sick steer was $-58. Our results are consistent with those of Brooks et al. [51], who found that the loss of net revenue per sick animal ranged from $60 to $143 and the net revenue provided by a sick animal ranged from $-61 to $-78. Poulsen Nautrup et al. [52] estimated the per case cost of BRD between $28 and $307 when prophylactic AMU was not administered.

In our model, the loss of net revenue induced by LA was $10, $25, and $41 per steer entering the feedlot for the carcass grades LA-, LA and LA+, respectively (see S1 Material). These consequences of LA are a sales price discount [31]. Data regarding the effects of LA are less frequent than for BRD. Though animals do not show clinical signs of LA unless severely affected, it has been previously reported that cattle presenting abscessed livers had a reduced ADG (up to 11%) and a lower feed efficiency [40]. This unavoidably increases the fattening duration and/or decreases the slaughter weight and devalues the quality grade [31].

To our knowledge, only Davis-Unger et al. [37] have calculated the economic impact of the different causes of lameness in beef feedlots. They calculated that each foot rot case cost $110 and each infectious arthritis case cost $727. The costs were due to decreased ADG, weight loss, and additional treatment costs. These results are consistent with our estimates ($152 for foot rot and $585 for infectious arthritis).

In the PCT scenario, the net revenue per steer entering the feedlot was $42. We observed that our results were sensitive to feed costs and sales prices. Time-series data show that indeed, this revenue from January 2016 to October 2018 varied from $-500 to $350 in Kansas and averaged $-40 [53].

While discussions regarding the efficacy and economics of preventing and controlling diseases such as liver abscesses and bovine respiratory disease focus on numeric comparisons, it is clear that there are stark differences in the perceived moral imperatives to use antibiotics for each indication [54]. Earlier work has shown a varying sense among feedlot veterinarians of the moral duty to use antibiotics to treat, control, and prevent disease. These differences grow when comparing to feedlot operators and to the general public. The immediacy of the need and the predictable differences in threats to animal welfare and mortality while preventing and controlling respiratory disease far outweigh perceptions of the importance of using medically important classes of antibiotic to prevent and control liver abscesses, which rarely exhibit animal welfare concerns.

Though AMU in animal agriculture is increasingly scrutinized, data reporting the economic implications of policies aiming at curbing AMU remain uncommon. In the beef, poultry and pig sectors, researchers have investigated the impact of banning growth promotion uses of medically important antimicrobials and concluded that such policies would have a minor to moderate impact [55–58]. It is noteworthy that since then, the U.S. government has fully implemented Guidance for Industry #209 and #213, banning the use of medically important AM as growth promoters in food animal production [59, 60]. In dairy production, Lhermie et al. estimated the average cost of entirely removing AM in the U.S. at $61 per cow, leading to a loss of $152 million for the U.S. dairy sector per annum [61, 62]. In the United States, there has been little movement towards banning various uses of antibiotic classes deemed to not be of importance to human medicine (e.g., ionophores, bacitracin, bambermycins). While the list derived from the FDA Guidance for Industry 152 [63] differs in modest ways from the list published and updated regularly by the WHO [64], the policy differences between, for example Europe, and the United States remain quite entrenched. Changes that occurred in the United States due to FDA GFI 209/213 mostly affected use of medically important antibiotics for growth promotion. Little activity has occurred since in changing, for example, labels that allow for continuous feeding with no defined duration. This seems to be the most likely next target in the United States and it is likely that products such as tylosin used to prevent/control liver abscesses would be required to be relabeled to better reflect the actual periods of risk for the development of abscessation.

In our feedlot model, removing prevention and control treatments induced a loss of $62 per steer entering the feedlot, under the scenario of moderate disease incidence. Our results are consistent with those from Dennis et al. [42], who report that the use of “upper tier” AM such as macrolides in prophylaxis allowed farmers to achieve a net revenue from $58 to $119 higher than if no prophylaxis was performed. In a meta-analysis, Abell et al. (2017), classified 8 AM used in prevention and control of BRD in lesser, middle, and upper tiers, in function of their odd-ratio, with “upper tier” AM being the most effective to decrease morbidity and mortality [7]. This additional net revenue ranged from $14 to $42 when “lower tier” AM such as sulfonamides or phenicols were used [42]. Prophylaxis was valued at 0.96% and 1.17% of the industry gross revenue, depending on the dataset. In a meta-analysis comparing conventional and nonconventional beef production, Wileman et al. [23] also reported that metaphylaxis enabled higher growth performance (0.11 kg/d of additional ADG) for the cattle treated. It is noteworthy that the existence of compensatory gain may moderate the ADG losses experienced during the clinical phase of disease, which are unlikely to be sustained through slaughter. We did not account for potential compensatory gains in our model.

Removing any kind of AMU led to negative revenue with moderate and high disease incidence under the assumptions of our model. When incidence rates were low, the net revenues were $27 and $12 per animal entering the feedlot, in the No-PC and No-PCT scenarios, respectively. This indicates that it seems feasible to achieve a positive net revenue even if drastic measures are taken concerning AMU in feedlots, but only if the incidence rates of major infectious diseases are low. This would require a large-scale adoption of non-medical and medical disease control strategies, such as preconditioning and changes in rations and feeding practices, as well as structural changes in the beef market [38, 65–67]. The effects of such management and structural changes were beyond the scope of our study and have not been incorporated in our model, but they may be worth investigating at the feedlot and supply chain levels.

One limitation of our model is that it does not account for animals dying from diseases without having been diagnosed, which may lead to an underestimation of the net impact. Our estimates do not include any type of aggregate market adjustment changes due to restrictions where lower production or higher costs might lead to higher finished beef prices. Our model was designed to provide information regarding short-run consequences of policies. This enables us to identify in detail which factors influence the loss of revenue, but does not model or estimate intermediate or long term impacts of policies, including the impact on other markets. Johnson et al. [68] estimated that reducing the prevalence of BRD would lead to an increased beef supply, and consequently losses of $2,904 million over 4 years for the beef industry [68].

Another limitation of our model is that we assumed that diseases were independent of one another. This hypothesis simplifies a very complex issue. Importantly, some diseases, such as BRD, LA and infectious arthritis, may be epidemiologically linked, and treatments may help control multiple diseases simultaneously [37, 69]. This may be associated with changes in animals’ immune status, as suggested by Duff and Galyean [70].

To assess how cow-calf producers might be affected in the long run, we calculated the decrease in purchase price (feeder cattle price) required to counterbalance the loss of net revenue in the No-PC and No-PCT scenarios. This presumes that those prices need to occur to maintain profitability in the feedlot. A decreased purchase price of 6.1% and 9%, for the No-PC and the No-PCT scenario respectively, allows the feedlot to maintain the revenue obtained in the PCT scenario. This would result in lower profits to beef cow producers who may respond by producing fewer calves resulting in higher feeder cattle prices. Another alternative for feedlot operators to maintain profitability would be to experience an increase in selling prices. Under moderate disease incidence rates, an increase in selling price of 4.2% and 6.3% in the No-PC and No-PCT scenario, respectively, enables the feedlot to maintain nearly the same expected revenue as in the PCT scenario. These increases in selling price is less than the average premium paid for organic products [71] and lower than twice the price of conventional beef that American consumers appear ready to spend for beef meat raised without AM and hormones, under supply and demand conditions existing at the time of the surveys. [72, 73].

Prohibition of AMU raises concerns of animal welfare. It is very likely that prohibition would worsen animal welfare on farms, as sick animals would remain untreated, culled or euthanized. Sixty-five percent of the conventional U.S. animal producers consider that raising animals without AM is a threat to animal welfare [74]. Animal welfare evaluation remains highly complex, as it takes multiple dimensions, and assessing the impacts of not using AM is needed prior to implementing restrictions. Antimicrobials have been proven effective to control infectious diseases, and remain a major tool in the therapeutic arsenal. Currently, few alternatives have emerged [75], but focusing on infection prevention and control, such as biosecurity measures, may help curb the need for AM.

In conclusion, our results suggest that the current beef feedlot sector would incur revenue losses, estimated between $43 and $139 per steer, in the case of restrictions on AMU. Policies should be designed to incentivize farmers to adopt non-AM preventive measures aimed at decreasing the need for AMU. Research for alternative methods to control bacterial diseases would also be valuable.

## Supporting information

S1 Material (DOCX)
