# Supplementary material for: Economic effects of policy options restricting antimicrobial use for high risk cattle placed in U.S. feedlots
Source: PLoS One. 2020 Sep 15;15(9):e0239135. doi: 10.1371/journal.pone.0239135 (PMC7491722; doi:10.1371/journal.pone.0239135)
Supplement: S1 Material — (DOCX) [file pone.0239135.s001.docx]

| Disease | Parameter | | | | | | |
| --- | --- | --- | --- | --- | --- | --- | --- |
|  | Dead | Realizer | Extra-DOF | Loss-QG | Additional  treatment cost | Additional  labor cost | Cost  adjustment |
| BRD-Cl1 | 4.68 | 3.40 | 0.36 | 0.89 | 1.29 | 0.08 | 1.84 |
| BRD-Cl2 | 1.17 | 0.73 | 0.00 | 0.22 | 0.32 | 0.02 | 0.26 |
| BRD-SubCl | 0.00 | 0.00 | 2.03 | 0.31 | 0.00 | 0.00 | 0.00 |
| LA- | 0.00 | 0.00 | 0.34 | 0.14 | 0.00 | 0.00 | 0.00 |
| LA | 0.00 | 0.00 | 0.52 | 0.07 | 0.00 | 0.00 | 0.00 |
| LA+ | 0.00 | 0.00 | 1.74 | 0.14 | 0.00 | 0.00 | 0.00 |
| Lame-FR | 0.13 | 0.03 | 0.01 | 0.02 | 0.01 | 0.00 | 0.02 |
| Lame-IA | 0.34 | 0.05 | 0.25 | 0.01 | 0.02 | 0.00 | 0.08 |
| Total | 6.32 | 4.21 | 5.24 | 1.80 | 1.65 | 0.10 | 2.20 |

**PCT, Low incidence**

| Disease | Parameter | | | | | | |
| --- | --- | --- | --- | --- | --- | --- | --- |
|  | Dead | Realizer | Extra-DOF | Loss-QG | Additional  treatment cost | Additional  labor cost | Cost  adjustment |
| BRD-Cl1 | 14.51 | 6.59 | 0.71 | 1.66 | 2.49 | 0.15 | 5.03 |
| BRD-Cl2 | 3.63 | 1.43 | 0.00 | 0.41 | 0.62 | 0.04 | 0.77 |
| BRD-SubCl | 0.00 | 0.00 | 4.19 | 0.59 | 0.00 | 0.00 | 0.00 |
| LA- | 0.00 | 0.00 | 1.36 | 0.51 | 0.00 | 0.00 | 0.00 |
| LA | 0.00 | 0.00 | 2.06 | 0.26 | 0.00 | 0.00 | 0.00 |
| LA+ | 0.00 | 0.00 | 6.90 | 0.51 | 0.00 | 0.00 | 0.00 |
| Lame-FR | 0.13 | 0.03 | 0.01 | 0.02 | 0.01 | 0.00 | 0.03 |
| Lame-IA | 0.34 | 0.05 | 0.27 | 0.01 | 0.02 | 0.00 | 0.09 |
| Total | 18.61 | 8.10 | 15.50 | 3.99 | 3.14 | 0.19 | 5.91 |

**No-PC, Low incidence**

| Disease | Parameter | | | | | | |
| --- | --- | --- | --- | --- | --- | --- | --- |
|  | Dead | Realizer | Extra-DOF | Loss-QG | Additional  treatment cost | Additional  labor cost | Cost  adjustment |
| BRD-Cl1 | 27.85 | 6.62 | 1.23 | 1.53 | 0.00 | 0.00 | 7.83 |
| BRD-Cl2 | 6.96 | 1.43 | 0.00 | 0.38 | 0.00 | 0.00 | 1.19 |
| BRD-SubCl | 0.00 | 0.00 | 5.27 | 0.59 | 0.00 | 0.00 | 0.00 |
| LA- | 0.00 | 0.00 | 2.48 | 0.51 | 0.00 | 0.00 | 0.00 |
| LA | 0.00 | 0.00 | 2.67 | 0.26 | 0.00 | 0.00 | 0.00 |
|  |  |  |  |  |  |  |  |
| LA+ | 0.00 | 0.00 | 7.76 | 0.51 | 0.00 | 0.00 | 0.00 |
| Lame-FR | 0.22 | 0.05 | 0.03 | 0.02 | 0.00 | 0.00 | 0.04 |
| Lame-IA | 0.48 | 0.06 | 0.23 | 0.01 | 0.00 | 0.00 | 0.12 |
| Total | 35.51 | 8.16 | 19.68 | 3.82 | 0.00 | 0.00 | 9.18 |

**No-PCT, Low incidence**

**Supp. table 1a:** **Additional costs and lost revenues per steer present in the feedlot at day 0, in low incidence. Extra-DOF: Additional Days On Feed; Loss-QG: Loss of Quality Grade; BRD-Cl1: Bovine Respiratory Disease with clinical signs occurring in Period 1; BRD-Cl2: Bovine Respiratory Disease with clinical signs occurring in Period 2; BRD-SubCl: Bovine Respiratory Disease with subclinical signs; LA-: Liver Abscess, mild intensity, LA: Liver Abscess, moderate intensity; LA+: Liver Abscess, severe intensity; Lame-FR: Foot rot; Lame-IA: Infectious Arthritis. PCT: Prevention Control and Treatment; No-PC: no Prevention, no Control; No-PCT: no Prevention, no Control and no Treatment.**

| Disease | Parameter | | | | | | |
| --- | --- | --- | --- | --- | --- | --- | --- |
|  | Dead | Realizer | Extra-DOF | Loss-QG | Additional  treatment cost | Additional  labor cost | Cost  adjustment |
| BRD-Cl1 | 8.77 | 6.38 | 0.67 | 1.67 | 2.42 | 0.14 | 3.44 |
| BRD-Cl2 | 2.19 | 1.37 | 0.00 | 0.42 | 0.61 | 0.04 | 0.49 |
| BRD-SubCl | 0.00 | 0.00 | 4.06 | 0.62 | 0.00 | 0.00 | 0.00 |
| LA- | 0.00 | 0.00 | 0.65 | 0.26 | 0.00 | 0.00 | 0.00 |
| LA | 0.00 | 0.00 | 1.35 | 0.19 | 0.00 | 0.00 | 0.00 |
| LA+ | 0.00 | 0.00 | 3.86 | 0.31 | 0.00 | 0.00 | 0.00 |
| Lame-FR | 1.67 | 0.42 | 0.06 | 0.21 | 0.18 | 0.03 | 0.28 |
| Lame-IA | 1.69 | 0.23 | 1.23 | 0.06 | 0.09 | 0.01 | 0.39 |
| Total | 14.33 | 8.40 | 11.89 | 3.73 | 3.29 | 0.22 | 4.60 |

**PCT, Moderate incidence**

| Disease | Parameter | | | | | | |
| --- | --- | --- | --- | --- | --- | --- | --- |
|  | Dead | Realizer | Extra-DOF | Loss-QG | Additional  treatment cost | Additional  labor cost | Cost  adjustment |
| BRD-Cl1 | 27.21 | 12.35 | 1.34 | 3.11 | 4.66 | 0.27 | 9.44 |
| BRD-Cl2 | 6.80 | 2.69 | 0.00 | 0.78 | 1.16 | 0.07 | 1.44 |
| BRD-SubCl | 0.00 | 0.00 | 8.39 | 1.19 | 0.00 | 0.00 | 0.00 |
| LA- | 0.00 | 0.00 | 2.57 | 0.97 | 0.00 | 0.00 | 0.00 |
| LA | 0.00 | 0.00 | 5.37 | 0.69 | 0.00 | 0.00 | 0.00 |
| LA+ | 0.00 | 0.00 | 15.33 | 1.14 | 0.00 | 0.00 | 0.00 |
| Lame-FR | 1.67 | 0.43 | 0.07 | 0.21 | 0.18 | 0.03 | 0.33 |
| Lame-IA | 1.69 | 0.23 | 1.34 | 0.06 | 0.09 | 0.01 | 0.43 |
| Total | 37.38 | 15.70 | 34.41 | 8.14 | 6.09 | 0.38 | 11.62 |

**No-PC, Moderate incidence**

| Disease | Parameter | | | | | | |
| --- | --- | --- | --- | --- | --- | --- | --- |
|  | Dead | Realizer | Extra-DOF | Loss-QG | Additional  treatment cost | Additional  labor cost | Cost  adjustment |
| BRD-Cl1 | 52.22 | 12.42 | 2.31 | 2.86 | 0.00 | 0.00 | 14.68 |
| BRD-Cl2 | 13.05 | 2.69 | 0.00 | 0.72 | 0.00 | 0.00 | 2.23 |
| BRD-SubCl | 0.00 | 0.00 | 10.54 | 1.19 | 0.00 | 0.00 | 0.00 |
| LA- | 0.00 | 0.00 | 4.68 | 0.97 | 0.00 | 0.00 | 0.00 |
| LA | 0.00 | 0.00 | 6.98 | 0.69 | 0.00 | 0.00 | 0.00 |
| LA+ | 0.00 | 0.00 | 17.25 | 1.14 | 0.00 | 0.00 | 0.00 |
| Lame-FR | 2.79 | 0.59 | 0.32 | 0.19 | 0.00 | 0.00 | 0.52 |
| Lame-IA | 2.38 | 0.30 | 1.17 | 0.05 | 0.00 | 0.00 | 0.59 |
| Total | 70.44 | 15.99 | 43.25 | 7.81 | 0.00 | 0.00 | 18.02 |

**No-PCT, Moderate incidence**

**Supp. table 1b:** **Additional costs and lost revenues per steer present in the feedlot at day 0, in moderate incidence. Extra-DOF: Additional Days On Feed; Loss-QG: Loss of Quality Grade; BRD-Cl1: Bovine Respiratory Disease with clinical signs occurring in Period 1; BRD-Cl2: Bovine Respiratory Disease with clinical signs occurring in Period 2; BRD-SubCl: Bovine Respiratory Disease with subclinical signs; LA-: Liver Abscess, mild intensity, LA: Liver Abscess, moderate intensity; LA+: Liver Abscess, severe intensity; Lame-FR: Foot rot; Lame-IA: Infectious Arthritis. PCT: Prevention Control and Treatment; No-PC: no Prevention, no Control; No-PCT: no Prevention, no Control and no Treatment.**

| Disease | Parameter | | | | | | |
| --- | --- | --- | --- | --- | --- | --- | --- |
|  | Dead | Realizer | Extra-DOF | Loss-QG | Additional  treatment cost | Additional  labor cost | Cost  adjustment |
| BRD-Cl1 | 13.45 | 9.78 | 1.03 | 2.56 | 3.72 | 0.22 | 5.28 |
| BRD-Cl2 | 3.36 | 2.10 | 0.00 | 0.64 | 0.93 | 0.05 | 0.75 |
| BRD-SubCl | 0.00 | 0.00 | 6.10 | 0.93 | 0.00 | 0.00 | 0.00 |
| LA- | 0.00 | 0.00 | 0.91 | 0.37 | 0.00 | 0.00 | 0.00 |
| LA | 0.00 | 0.00 | 2.03 | 0.28 | 0.00 | 0.00 | 0.00 |
| LA+ | 0.00 | 0.00 | 8.49 | 0.68 | 0.00 | 0.00 | 0.00 |
| Lame-FR | 14.45 | 3.64 | 0.55 | 1.80 | 1.52 | 0.26 | 2.44 |
| Lame-IA | 23.72 | 3.22 | 17.26 | 0.79 | 1.21 | 0.14 | 5.42 |
| Total | 54.98 | 18.75 | 36.36 | 8.05 | 7.38 | 0.67 | 13.89 |

**PCT, High incidence**

| Disease | Parameter | | | | | | |
| --- | --- | --- | --- | --- | --- | --- | --- |
|  | Dead | Realizer | Extra-DOF | Loss-QG | Additional  treatment cost | Additional  labor cost | Cost  adjustment |
| BRD-Cl1 | 41.73 | 18.94 | 2.05 | 4.77 | 7.14 | 0.42 | 14.47 |
| BRD-Cl2 | 10.43 | 4.12 | 0.00 | 1.19 | 1.79 | 0.10 | 2.20 |
| BRD-SubCl | 0.00 | 0.00 | 12.58 | 1.78 | 0.00 | 0.00 | 0.00 |
| LA- | 0.00 | 0.00 | 3.63 | 1.37 | 0.00 | 0.00 | 0.00 |
| LA | 0.00 | 0.00 | 8.05 | 1.03 | 0.00 | 0.00 | 0.00 |
| LA+ | 0.00 | 0.00 | 33.74 | 2.52 | 0.00 | 0.00 | 0.00 |
| Lame-FR | 14.45 | 3.72 | 0.62 | 1.80 | 1.52 | 0.26 | 2.82 |
| Lame-IA | 23.72 | 3.25 | 18.77 | 0.79 | 1.21 | 0.14 | 5.96 |
| Total | 90.32 | 30.03 | 79.44 | 15.26 | 11.66 | 0.92 | 25.45 |

**No-PC, High incidence**

| Disease | Parameter | | | | | | |
| --- | --- | --- | --- | --- | --- | --- | --- |
|  | Dead | Realizer | Extra-DOF | Loss-QG | Additional  treatment cost | Additional  labor cost | Cost  adjustment |
| BRD-Cl1 | 80.07 | 19.04 | 3.54 | 4.39 | 0.00 | 0.00 | 22.51 |
| BRD-Cl2 | 20.02 | 4.12 | 0.00 | 1.10 | 0.00 | 0.00 | 3.42 |
| BRD-SubCl | 0.00 | 0.00 | 15.82 | 1.78 | 0.00 | 0.00 | 0.00 |
| LA- | 0.00 | 0.00 | 6.61 | 1.37 | 0.00 | 0.00 | 0.00 |
| LA | 0.00 | 0.00 | 10.46 | 1.03 | 0.00 | 0.00 | 0.00 |
| LA+ | 0.00 | 0.00 | 37.95 | 2.52 | 0.00 | 0.00 | 0.00 |
| Lame-FR | 24.18 | 5.09 | 2.79 | 1.68 | 0.00 | 0.00 | 4.48 |
| Lame-IA | 33.26 | 4.19 | 16.42 | 0.69 | 0.00 | 0.00 | 8.25 |
| Total | 157.52 | 32.45 | 93.59 | 14.55 | 0.00 | 0.00 | 38.66 |

**No-PCT, High incidence**

**Supp. table 1c:** **Additional costs and lost revenues per steer present in the feedlot at day 0, in high incidence. Extra-DOF: Additional Days On Feed; Loss-QG: Loss of Quality Grade; BRD-Cl1: Bovine Respiratory Disease with clinical signs occurring in Period 1; BRD-Cl2: Bovine Respiratory Disease with clinical signs occurring in Period 2; BRD-SubCl: Bovine Respiratory Disease with subclinical signs; LA-: Liver Abscess, mild intensity, LA: Liver Abscess, moderate intensity; LA+: Liver Abscess, severe intensity; Lame-FR: Foot rot; Lame-IA: Infectious Arthritis. PCT: Prevention Control and Treatment; No-PC: no Prevention, no Control; No-PCT: no Prevention, no Control and no Treatment.**

| Disease | Costs of  an individual case |
| --- | --- |
| BRD-Cl1 | $138.44 |
| BRD-Cl2 | $137.75 |
| BRD-SubCl | $23.41 |
| LA- | $10.69 |
| LA | $25.61 |
| LA+ | $41.67 |
| Lame-FR | $152.20 |
| Lame-IA | $584.65 |

**Supp. Table 2: costs estimates per case simulated in the model.**

**Extra-DOF: Additional Days On Feed; Loss-QG: Loss of Quality Grade; BRD-Cl1: Bovine Respiratory Disease with clinical signs occurring in Period 1; BRD-Cl2: Bovine Respiratory Disease with clinical signs occurring in Period 2; BRD-SubCl: Bovine Respiratory Disease with subclinical signs; LA-: Liver Abscess, mild intensity, LA: Liver Abscess, moderate intensity; LA+: Liver Abscess, severe intensity; Lame-FR: Foot rot; Lame-IA: Infectious Arthritis. PCT: Prevention Control and Treatment; No-PC: no Prevention, no Control; No-PCT: no Prevention, no Control and no Treatment.**

| Parameter | Distribution  modelled | Min | Mean | Max | 5% | 95% |
| --- | --- | --- | --- | --- | --- | --- |
| Treatments costs ($) | Triangular | 10.03 | 12.11 | 13.68 | 10.69 | 13.25 |
| Feed costs ($) | Triangular | 1.18 | 1.84 | 2.73 | 1.36 | 2.44 |
| Slaughter cattle price ($) | Normal | 1.84 | 2.60 | 3.37 | 2.27 | 2.93 |
| Feeder cattle price ($) | Normal | 2.33 | 3.44 | 4.62 | 2.95 | 3.93 |
| BRD RR-MORB | Triangular | 0.45 | 0.52 | 0.59 | 0.47 | 0.57 |
| LA RR-MORB | Triangular | 0.23 | 0.27 | 0.33 | 0.24 | 0.31 |
| BRD RR-FAT | Triangular | 0.50 | 0.63 | 0.76 | 0.54 | 0.72 |
| Extra DOF | Triangular | 6.2% | 7.5% | 9.1% | 6.6% | 8.6% |

**Supp. Table 3: Parameters’ distribution values simulated in the model.**

**BRD RR-MORB: relative risk of Bovine Respiratory Disease morbidity when antimicrobials are not used for prevention. LA RR-MORB: relative risk of liver abscesses morbidity when antimicrobials are not used for prevention; BRD RR-FAT: relative risk of Bovine Respiratory Disease fatality when antimicrobials are not used for prevention; Extra-DOF: Additional Days On Feed.**
